# Implementation strategies to increase Malawian health care workers’ knowledge about and self-efficacy to recommend HPV vaccination: A pilot study

**DOI:** 10.1371/journal.pgph.0006508

**Published:** 2026-05-19

**Authors:** Corrina Moucheraud, Sam Phiri, Khumbo Phiri, Pericles Kalande, Amos Makwaya, Linda Kamtsendero, Harrison Chimbaka, Nükte Göç Gürpınar, Christine Hagstrom, Joep J. van Oosterhout, Peter G. Szilagyi, Kate Guastaferro, Risa M. Hoffman

**Affiliations:** 1 Department of Public Health Policy and Management, School of Global Public Health, New York University, New York, United States of America; 2 Partners in Hope, Lilongwe, Malawi; 3 Department of Medicine, David Geffen School of Medicine, University of California Los Angeles, Los Angeles, California, United States of America; 4 Department of Pediatrics, David Geffen School of Medicine, University of California Los Angeles, Los Angeles, California, United States of America; 5 Department of Social and Behavioral Sciences, School of Global Public Health, New York University, New York, United States of America; PLOS: Public Library of Science, UNITED STATES OF AMERICA

## Abstract

Health workers’ recommendation of HPV vaccination is a highly effective evidence-based intervention for increasing the uptake of this important cancer prevention tool. In Malawi, as in many countries, HPV vaccine uptake is low. In preparation for an implementation science study using an intervention optimization design, we pilot tested two implementation strategies aimed at increasing Malawian health workers’knowledge and self-efficacy to counsel about HPV vaccination. Health workers were recruited from six large health facilities in Southern Malawi; each participant took a brief assessment before and after viewing the pilot strategy: either a training (aimed to increase knowledge) or video vignettes of counseling discussions (to increase self-efficacy to counsel). There were 45 health workers who participated, 19 attended the training, and 26 viewed the videos. Knowledge increased approximately 20 points on average (0–100 scale), and knowledge increases were greater among health workers who participated in the training (24.1 points versus 16.5 points for those who watched the videos). Self-efficacy increased 13.7 points on average (0–100 scale) and to a greater extent among those who viewed the videos (14.2 point increase) than those who attended the training (13.1 point increase). Both training and videos were rated very high for acceptability. These results suggest that brief training and video vignettes may be effective and offer complementary benefits for increasing health workers’ knowledge and self-efficacy to counsel about HPV vaccination. We will next test these strategies in a large cluster randomized controlled trial using the multi-phase optimization strategy to identify the most effective and implementable set of implementation strategies for increasing health worker recommendation of, and ultimately coverage of, the HPV vaccine in Malawi.

## Introduction

Vaccination against human papillomavirus (HPV) is an important tool in the global effort to eliminate cervical cancer [1–3], especially in countries like Malawi, which has the second-highest cervical cancer incidence and mortality globally [4] and challenges in delivering cancer screening at scale [5]. Malawi introduced the HPV vaccine in 2019 [6], and eligible girls (9–14 years) can receive the vaccine free of charge at schools, health facilities, and in communities. However, only 13% of eligible Malawian girls received any HPV vaccine doses in 2021 and 2022 [7]. Other countries face a similar challenge [7,8].

There are many reasons for low uptake of the HPV vaccine globally; our team has identified numerous important intra- and inter-personal factors that may shape parents’ decisions about whether to vaccinate their daughters, including attitudes about the HPV vaccine, perceiving pro-HPV vaccination social norms, and speaking to other people about HPV vaccination – including health workers [9,10]. Malawian girls whose parents who had spoken with a health worker about HPV vaccination had nearly three times the adjusted odds of having received the HPV vaccine than their peers whose parents had not had such a conversation [10]. A highly effective evidence-based intervention to increase HPV vaccination is health worker recommendation [11] – but there is limited evidence from lower-income countries about how to encourage health workers to recommend the HPV vaccine.

Our team is undertaking a multi-year study to develop, implement, and evaluate implementation strategies to increase health worker recommendation of HPV vaccination to girls and young women with HIV in Malawi, who are a priority group for HPV vaccination due to their high risk for cervical cancer [12–14]. Following the multi-phase optimization strategy (MOST) paradigm [15,16], this study (“Kupewa,” which means “prevent” in the Chichewa language of Malawi) will evaluate the effectiveness and implementability of three implementation strategies, alone and in combination.

Intervention optimization is a deliberate process to evaluate multi-component interventions (here, implementation strategies [17]) by balancing effectiveness against other outcomes like affordability or scalability. MOST is a specific approach to intervention optimization that involves three distinct phases: preparation (including identifying, designing, and pilot testing the components), optimization (evaluate the components alone and in combination, using an optimization randomized controlled trial design), and evaluation (use a traditional two-armed trial design to assess effectiveness of the optimized multi-component approach) [18].

Here we present results from the pilot (“preparation”) phase of the Kupewa study, which was designed to apply MOST to implementation strategies for increasing HPV vaccination in Malawi. The goal of the preparation phase of MOST is to ensure that the strategies are implementable and show promise for a subsequent optimization trial [19]. Thus, in this pilot study, we sought to ascertain if knowledge and self-efficacy in skills to counsel about HPV vaccination increased following exposure to implementation strategies designed to target these hypothesized determinants of health worker recommendation.

## Materials and methods

### Ethics statement

This study was reviewed and approved by the Malawi National Health Sciences Research Committee (protocol #24/03/4399) and the New York University Institutional Review Board (protocol # FY2024–8481). All participants provided written informed consent prior to the intervention. Additional information regarding the ethical, cultural, and scientific considerations specific to inclusivity in global research is included in S2 File.

### Study overview

The study presented here aimed to develop and pilot test two implementation strategies to increase health workers’ recommendation of HPV vaccination to eligible girls and young women living with HIV in Malawi. This pilot study was the first step toward a full randomized controlled trial to evaluate these strategies alone and in combination. See S3 File for more information.

### Intervention

Informed by the Capabilities-Opportunity-Motivation-Behavior (COM-B) framework [20–23], we designed three specific implementation strategies to target factors hypothesized to be associated with whether a health worker recommends the HPV vaccine to their clients: their *knowledge* about the HPV vaccination (targeted by a training), their *skills to counsel about HPV vaccination* (targeted by video vignettes), and *lack of clarity about which girls are eligible* (targeted by a reminder/prompt system). In this pilot study, we focus on the training and video strategies, as our team already has extensive familiarity with the feasibility of reminder/prompt systems in this context. The intervention was designed specifically for health workers in Malawi who provide care and treatment to girls living with HIV, as this is a high-priority group for vaccination due to their elevated risk for cervical cancer.

The one-hour, facility-based trainings were led by a clinical expert in cervical cancer prevention and HPV vaccination. The training used a didactic format – with PowerPoint slides adapted from tools used by the World Health Organization and incorporating inputs from the Malawi Ministry of Health Reproductive Health Directorate – followed by brief questions and answers with the trainer. Topics included the epidemiology of HPV and cervical cancer; HPV vaccination eligibility in Malawi; dosage schedule, contraindications, and side effects; and suggestions about how to recommend the HPV vaccine following a modified version of the “five As” health behavior counseling approach [24–26] advocated by the World Health Organization for vaccination communication: advise on the vaccine and schedule, alert on side effects, and arrange for when to return for remaining doses.

The videos were developed by our study team and included the same information as the training, but delivered as a conversation between a health worker and a parent or young woman using best practices about making an HPV vaccination recommendation [27–29]. Actors were used to create five professionally- recorded and- edited videos. During post-production, we added a voiceover and subtitles highlighting best practices. Each video was between 13 and 20 minutes in duration. Participating health workers watched all five videos individually (on a tablet using headphones) at their own pace over a one-hour period. There was no discussion or question/answer period in this intervention.

### Sample and recruitment

This pilot study was conducted at six health facilities in southern Malawi. These facilities were selected because they serve large numbers of girls and young women living with HIV. The six facilities were randomly allocated 1:1 to receive either the training or the videos; random numbers were generated in Excel to determine assignment. Between 6/August/2024 and 12/August/2024, at each of the selected sites, a member of the study team introduced the pilot study to the facility in- charge, and invited health workers to attend the lunchtime pilot session that same day (and that day only).

### Data collection

We developed a short survey to assess the knowledge and self-efficacy of health workers’ skills to counsel about HPV vaccination (S1 File). The survey was administered before and after viewing either the training or the videos, depending on site assignment (i.e., pre-test/post-test). The survey incorporated 14 true/false or multiple choice questions reflecting the items discussed in the training intervention (“knowledge” items), and 12 “self-efficacy” questions adapted from the self-efficacy questionnaire for measuring health workers’ clinical communication skills (SE-12; each rated on a 10-point scale) [30]. The post-test survey also included questions about the acceptability and feasibility of the intervention based on the Training Acceptability Rating Scale [31]. We also collected basic information about the respondents (e.g., age, gender, cadre). All health workers provided written informed consent, then self-administered the pre-test using tablet-based data collection with SurveyCTO software, then engaged in the intervention to which the facility was assigned (either training or videos), and lastly self-administered the post-test survey. Health workers were provided a lunch allowance for participation and refreshments. The data are available at https://doi.org/10.58153/bp0pk-te632 [32].

### Data analysis

We calculated knowledge and skills’ self-efficacy in the pre-test and the post-test per item and as an overall score, which was a sum of individual items that we standardized to a 0–100 scale. We hypothesized that changes in knowledge would be greater among health workers who participated in the training, and changes in skills’ self-efficacy would be greater among health workers who viewed the videos.

## Results

In total, 45 health workers participated in this pilot study: 19 participated in the training, and 26 viewed the videos (see S1 Table for sample characteristics).

**Fig 1:**
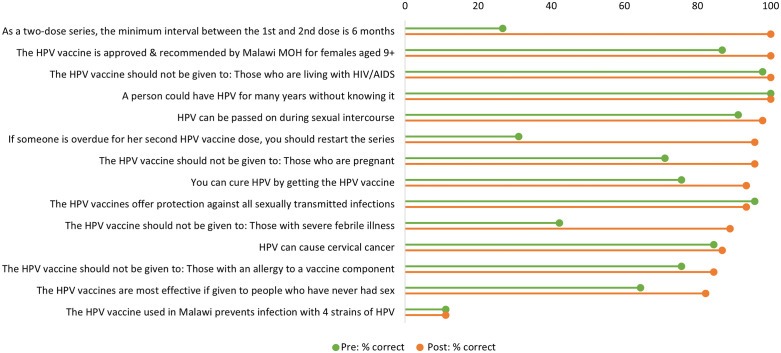
Knowledge item changes from pre- to post-test (n=45).

Knowledge improved on 11 of the 14 items from pre- to post- test (Fig 1). The biggest increases were seen for items that reflected specific clinical knowledge, for example, “As a two-dose series, the minimum interval between the 1st and 2nd dose is 6 months,” which was answered correctly by only 26.7% of respondents during the pre-test and by 100% in the post-test (Fig 1). All health workers responded correctly to “A person could have HPV for many years without knowing it” during the pre- and post- tests, so there was no improvement on this item, and over 90% responded correctly to “The HPV vaccines offer protection against all sexually transmitted infections” at both pre- and post-test (Fig 1).

Overall knowledge scores increased from an average of 68.1 points to 87.8 points (Table 1). At the sites where the training was piloted, health workers’ knowledge scores increased on average by 24.1 points (from 67.3 points to 91.4 points); at the sites where videos were piloted, the corresponding increase in knowledge scores was 16.5 points (from 68.8 points to 85.2 points).

**Table 1:** Outcomes at pre-test and at post-test, overall and by intervention assignment.

	Pre score, mean (standard deviation)	Post score, mean (standard deviation)
**KNOWLEDGE**		
At all sites (n = 45)	68.1 (11.6)	87.8 (8.1)
At training sites (n = 19)	67.3 (12.9)	91.4 (5.6)
At video sites (n = 26)	68.7 (10.7)	85.2 (8.8)
**SKILLS’ SELF-EFFICACY**		
At all sites (n = 45)	82.6 (15.4)	96.4 (5.4)
At training sites (n = 19)	83.4 (15.2)	96.5 (4.3)
At video sites (n = 26)	82.1 (15.8)	96.3 (6.2)

*Knowledge and self-efficacy scores could range from 0-100 each.*

**Fig 2:**
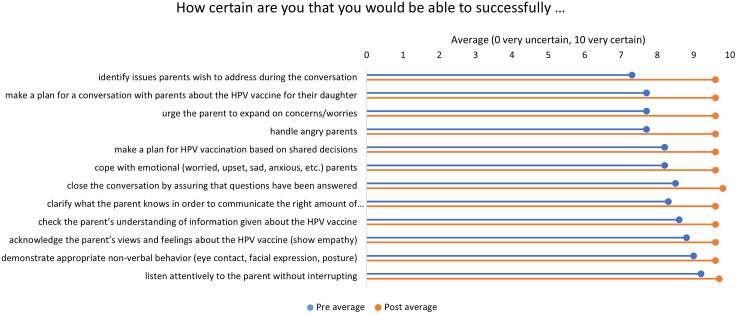
Self-efficacy in counseling skills item changes from pre- to post-test (n = 45).

Self-efficacy in counseling skills increased on all items from pre- to post-test (Fig 2). The largest increases were in being able to identify issues to discuss (increased by 31.5%), and making a plan for the conversation, urging parents to expand on their concerns, and handling angry parents (all increased by 24.7%).

Health workers reported high self- efficacy, with an average pre-test self-efficacy score of 82.6 and an average post-test score of 96.4. The score increased following exposure to the pilot intervention by 13.7 points on average across all sites (Table 1). The increase was greater at video sites (14.2 points on average) than at training sites (13.1 points on average).

We found very high acceptability of the training and the videos: nearly every element received a perfect score of 4 (possible range 1–4) (S2 Table).

## Discussion

In this pilot study, these implementation strategies increased health workers’ knowledge about HPV vaccination and increased their self-efficacy in skills to discuss HPV vaccination with girls and their parents. The findings provide strong support for the Kupewa study, which will formally evaluate whether these strategies change health workers’ behavior– i.e., if these increases in knowledge and self-efficacy translate to increased recommendation of HPV vaccination to girls and young women living with HIV as hypothesized, and if this results in greater vaccine uptake among these girls and young women.

We found greater increases in knowledge among health workers who received the training intervention, and greater increases in self-efficacy among those who watched the videos. These differential changes correspond to the underlying constructs from COM-B that we hypothesized would respond to these specific activities.

In our sample, health workers lacked specific knowledge about HPV vaccination, despite being well-informed about the public health-related aspects of HPV and cervical cancer. Knowledge gaps mostly reflected clinical details (timing between doses, contraindications, etc.). This suggests the importance of training as part of an intervention to improve provider recommendation for HPV vaccination. Our full trial will evaluate training alone and in combination with video vignettes to determine which combination most increases health workers’ recommendation of HPV vaccination during discussions with girls (and parents) and young women.

Health workers generally reported high self-efficacy in counseling skills during the pre-test, but there were large improvements in thoughtfully engaging parents in the conversation and being proactive in steering the discussion, even when difficult. Given common concerns about HPV vaccine safety in Malawi [9], these more advanced counseling skills will likely be critical to achieve widespread vaccine uptake.

Some limitations should be noted. First, as this was a pilot study, it was not designed or intended to detect statistically significant differences; we therefore did not conduct these tests, but subsequent evaluation studies should. Second, because this was a pilot study, results may not generalize to other contexts. A larger study would also permit subgroup comparisons (e.g., cadres or duration of job experience). Related, we did not collect any data about who participated in the pilot study at these sites versus who did not, so we cannot assess whether there are any unique attributes of participants owing to potential selection bias. Third, knowledge of certain topics was near-universal during the pre-test, and pre-test self-efficacy was high for many items, resulting in ceiling effects. Likewise, nearly all participants reported the maximum acceptability scores for all items, suggesting that improved implementation outcome measures may be warranted.

## Conclusions

Implementation strategies aiming to increase Malawian health workers’ knowledge and self-efficacy to counsel may be implementable and may result in complementary improvements. Our team will next evaluate whether these strategies increase health workers’ recommendation of HPV vaccination and HPV vaccine uptake among girls/young women living with HIV in Malawi through an optimization trial in a study informed by the MOST framework.

## Supporting information

S1 File Pre- and post-test survey items. (DOCX)

S2 File PLoS questionnaire on inclusivity in global research. (DOCX)

S3 File CONSORT 2010 checklist, pilot or feasibility trial (Eldridge SM, Chan CL, Campbell MJ, Bond CM, Hopewell S, Thabane L, et al. CONSORT 2010 statement: extension to randomised pilot and feasibility trials. BMJ. 2016;355.).
(DOC)

S1 Table Sample characteristics. (DOCX)

S2 Table Acceptability of pilot intervention. (DOCX)
