# Supplementary material for: Implementation strategies to increase Malawian health care workers’ knowledge about and self-efficacy to recommend HPV vaccination: A pilot study
Source: PLOS Glob Public Health. 2026 May 19;6(5):e0006508. doi: 10.1371/journal.pgph.0006508 (PMC13186351; doi:10.1371/journal.pgph.0006508)
Supplement: S1 File — (DOCX) [file pgph.0006508.s001.docx]

**S1 Text: Pre- and post-test survey items**

1. **Knowledge** (each True / False / I don’t know unless otherwise indicated):

- HPV can cause cervical cancer
- HPV can be passed on during sexual intercourse
- A person could have HPV for many years without knowing it
- The HPV vaccine requires at least 2 doses
- The HPV vaccines offer protection against all sexually transmitted infections
- The HPV vaccines are most effective if given to people who have never had sex
- The HPV vaccine should not be given to those with an allergy to a vaccine components
- The HPV vaccine should not be given to those who are pregnant
- The HPV vaccine should not be given to those with severe febrile illness
- The HPV vaccine should not be given to those who are living with HIV/AIDS
- If someone is overdue for her second HPV vaccine dose, you should restart the series
- You can cure HPV by getting the HPV vaccine
- The HPV vaccine is approved and recommended by the Malawi Ministry of Health for females aged ____ years and older (Response options: 7 / 9 / 11 / I don’t know)
- The HPV vaccine used in Malawi prevents infection with ___ strains of HPV (Response options: 1 / 2 /4 / I don’t know)
- When giving HPV vaccine as a two-dose series, the minimum interval between the first and second dose is __ months (Response options: 1 / 3 / 6 / 12 / I don’t know)

1. **Self-efficacy in counseling skills** (each 1-10 point scale where 1 is “Very uncertain” and 10 is “Very certain”)

- How certain are you that you would be able to successfully identify the issues parents wish to address during a conversation about the HPV vaccine for their daughter?
- How certain are you that you would be able to successfully make a plan for a conversation with parents about the HPV vaccine for their daughter?
- How certain are you that you would be able to successfully urge the parent to expand on his or her concerns/worries about the HPV vaccine for their daughter?
- How certain are you that you would be able to successfully listen attentively to the parent without interrupting?
- How certain are you that you would be able to successfully demonstrate appropriate non-verbal behavior (eye contact, facial expression, placement, posture, and voicing) during a conversation with a parent about the HPV vaccine for their daughter?
- How certain are you that you would be able to successfully show empathy (acknowledge the parent’s views and feelings about the HPV vaccine for their daughter)?
- How certain are you that you would be able to successfully clarify what the parent knows about the HPV vaccine in order to communicate the right amount of information?
- How certain are you that you would be able to successfully check the parent’s understanding of the information given about the HPV vaccine?
- How certain are you that you would be able to successfully make a plan for HPV vaccination for their daughter based on shared decisions between you and the parent?
- How certain are you that you would be able to successfully close the conversation by assuring that the parent’s questions about the HPV vaccine have been answered?
- How certain are you that you would be able to successfully cope with emotional parents?
- How certain are you that you would be able to successfully handle angry parents?
