# Supplementary material for: Implementation strategies to increase Malawian health care workers’ knowledge about and self-efficacy to recommend HPV vaccination: A pilot study
Source: PLOS Glob Public Health. 2026 May 19;6(5):e0006508. doi: 10.1371/journal.pgph.0006508 (PMC13186351; doi:10.1371/journal.pgph.0006508)
Supplement: S2 File — (DOCX) [file pgph.0006508.s002.docx]

Inclusivity in global research

**Ethical considerations, permits and authorship**

*This section is applicable to all research types.*

Provide details as to who granted permissions and/or consent for the study to take place in the Methods section of your manuscript. This should include the names of **all** ethics boards, governmental organizations, community leaders or other bodies that provided approval for the study. If individuals provided approval refer to these people by their role or title but do not list their name(s).

Reported on page number: 7

If there were any deviations from the study protocol after approval was obtained please provide details of these changes in the Methods section of your manuscript.

n/a (no deviations)

Did this study involve local collaborators that are residents of the country where the research was conducted or members of the community studied? If you do not have any authors from said communities, please provide an explanation for this below.

Yes

Everyone listed as an author should meet PLOS’ criteria for authorship and all individuals who meet these criteria should be included in the author byline, rather than the acknowledgements. For further information please see the journal’s Authorship Policy.

Yes

**Human subjects research (e.g. health research, medical research, cross-cultural psychology)**

Did you obtain written informed consent from a representative of the local community or region before the research took place? How did you establish who speaks for the community? Details of written informed consent obtained from study participants should be reported separately in the Methods section of your manuscript.

Yes written informed consent was obtained from all study participants (page 7).

How did members of the local community provide input on the aims of the research investigation, its methodology, and its anticipated outcome(s)? When engaging with the local community, how did you ensure that the informed consent documents and other materials could be understood by local stakeholders?

This study was designed and implemented in full cooperation with researchers in Malawi. The study protocol was also reviewed and approved by both ethical and scientific review committees in Malawi, both at the national and district levels. All study materials (informed consent, survey tools) were developed and implemented in English language as this is standard for communicating with and among health workers in this setting. We ensured the tools were understandable through pretesting.

Will the findings of the research be made available in an understandable format to stakeholders in the community where the study was conducted (e.g. via a presentation, summary report, copies of publications, etc.)? Please provide details of how this will be achieved.

Yes, findings have been disseminated with key stakeholders within the Ministry of Health and otherwise in Malawi and will be shared with the District teams who are then responsible for coordinating results dissemination activities at relevant health facilities.
