# Supplementary material for: Implementation strategies to increase Malawian health care workers’ knowledge about and self-efficacy to recommend HPV vaccination: A pilot study
Source: PLOS Glob Public Health. 2026 May 19;6(5):e0006508. doi: 10.1371/journal.pgph.0006508 (PMC13186351; doi:10.1371/journal.pgph.0006508)
Supplement: S1 Table — (DOCX) [file pgph.0006508.s004.docx]

**S1 Table**: Sample characteristics

| Gender | Female | 25 |
| --- | --- | --- |
|  | Male | 20 |
| Cadre | Clinical officer | 7 |
|  | Nurse | 30 |
|  | Medical assistant | 7 |
|  | Other (community midwife) | 1 |
|  | Years of experience in this role (avg. median) | 8.3 (5) |
