# Supplementary material for: Implementation strategies to increase Malawian health care workers’ knowledge about and self-efficacy to recommend HPV vaccination: A pilot study
Source: PLOS Glob Public Health. 2026 May 19;6(5):e0006508. doi: 10.1371/journal.pgph.0006508 (PMC13186351; doi:10.1371/journal.pgph.0006508)
Supplement: S2 Table — (DOCX) [file pgph.0006508.s005.docx]

**S2 Table**: Acceptability of pilot intervention

|  | Training sites, average score (range) | Video sites, average score (range) |
| --- | --- | --- |
| Did the pilot intervention improve your understanding of the HPV vaccine? | 4 (4-4) | 3.9 (3-4) |
| Did the pilot intervention help you to develop skills to discuss the HPV vaccine with parents? | 4 (4-4) | 3.9 (3-4) |
| Did the pilot intervention make you more confident in discussing the HPV vaccine with parents? | 3.9 (3-4) | 4.0 (3-4) |
| Do you expect to make use of what you learned in your role as a health worker? | 3.9 (3-4) | 3.9 (3-4) |
| Did the pilot intervention cover the topics you feel are important related to the HPV vaccine? | 3.9 (3-4) | 3.9 (3-4) |
| In an overall/general sense, how satisfied are you with the pilot intervention? | 3.8 (3-4) | 3.9 (3-4) |
